# Association between residents’ work hours and patient care ownership: a nationwide cross-sectional study in Japan

**DOI:** 10.1186/s12909-025-06941-1

**Published:** 2025-03-15

**Authors:** Hirohisa Fujikawa, Hidetaka Tamune, Yuji Nishizaki, Kazuya Nagasaki, Hiroyuki Kobayashi, Masanori Nojima, Miwa Sekine, Taro Shimizu, Yu Yamamoto, Kiyoshi Shikino, Yasuharu Tokuda

**Affiliations:** 1 Center for General Medicine Education, School of Medicine, Keio University, 35 Shinanomachi, Shinjuku-ku, Tokyo, 160- 8582 Japan; 2 Department of Medical Education Studies, International Research Center for Medical Education, Graduate School of Medicine, The University of Tokyo, Bunkyo-ku, Tokyo, Japan; 3 Present Address: Department of General Medicine, Juntendo University Faculty of Medicine, Bunkyo-ku, Tokyo, Japan; 4 Department of Psychiatry and Behavioral Science, Juntendo University Graduate School of Medicine, Bunkyo-ku, Tokyo, Japan; 5 Division of Medical Education, Juntendo University School of Medicine, Bunkyo-ku, Tokyo, Japan; 6 Department of Internal Medicine, Mito Kyodo General Hospital, University of Tsukuba, Mito, Ibaraki Japan; 7 Center for Translational Research, The Institute of Medical Science Hospital, The University of Tokyo, Minato-ku, Tokyo, Japan; 8 Department of Diagnostic and Generalist Medicine, Dokkyo Medical University Hospital, Mibu-machi, Shimotsuga-gun, Tochigi Japan; 9 Division of General Medicine, Center for Community Medicine, Jichi Medical University, Shimotsuke, Tochigi Japan; 10 Department of Community-Oriented Medical Education, Graduate School of Medicine, Chiba University, Chiba, Chiba Japan; 11 Muribushi Okinawa for Teaching Hospitals, Urasoe, Okinawa Japan; 12 Tokyo Foundation for Policy Research, Minato-ku, Tokyo, Japan

**Keywords:** Professionalism, Workplace conditions, Patient care, Internship and residency

## Abstract

**Purpose:**

In the current era of physician work-hour regulations, patient care ownership (PCO) has received considerable attention. The aim of the study was to investigate the association between working hours and PCO.

**Methods:**

This was a nationwide cross-sectional study. The study recruited residents who completed the General Medicine In-Training Examination. The primary outcome was PCO, assessed using the Japanese version of the PCO Scale (J-PCOS). The secondary outcomes were the four dimensions of the J-PCOS (i.e., assertiveness, sense of ownership, diligence, and being the “go-to” person). The explanatory variable was weekly working hours. We examined the association using multivariable linear regression analysis.

**Results:**

1836 participants were included in the analysis. After adjustment for possible confounders, residents working ≥ 70 to < 90 h/week had greater PCO than those working ≥ 60 to < 70 h/week. Working ≥ 70 to < 90 h/week was also associated with assertiveness and being the “go-to” person. No clear trend was seen in the relationship between working hours and sense of ownership or diligence.

**Conclusion:**

While determining appropriate resident work hours requires comprehensive consideration of a number of factors, in terms of PCO training, the working hours of 80–90 h/week may be an option.

**Supplementary Information:**

The online version contains supplementary material available at 10.1186/s12909-025-06941-1.

## Background

Long working hours are an issue of global concern in the medical field [1, 2]. Medical residents in particular are exposed to long work hours [3]. Numerous studies have shown negative effects of such long work hours, including poorer physician health and a lower quality of patient care [4–6]. As a result, many countries have implemented duty hour restrictions for medical residents in recent decades [7]. Also in Japan, extended working hours have long been a problem for physicians [8], and working hour regulations were finally introduced in April 2024 [9]. Under these regulations, physicians are allowed to work up to 960 h of overtime per year (equivalent to a 60-hour work week), with some exceptions (e.g., medical trainees in high-intensity training) [9, 10].

Residency duty hour policies are continually assessed and modified out of concern for patient safety and resident well-being [11]. Numerous studies have examined the effects of resident working hour regulations on various outcomes. For medical resident mental health outcomes, a recent systematic review and meta-analysis found that shorter work hours were positively associated with improved mental condition (i.e., less burnout and better well-being) [11]. Conversely, for resident education, few studies have examined meaningful educational outcomes [12]. For example, while many researchers and clinical educators around the world have expressed concern about the impact of working hour restrictions on the medical professionalism of residents and its component, patient care ownership (PCO) [13–15], few studies have actually examined these outcomes [16].

PCO – a crucial component of medical professionalism [17] – is defined as the cognitive-affective state that develops as a result of the individual’s knowledge of patients, their management, and the emotional investment in the relationship with them [15, 17, 18]. PCO is important for delivering quality patient care [15, 18]. For medical trainees, the nurturing of PCO is expected to improve clinical skills and patient outcomes [17]. PCO consists of multiple constructs, including physician actions (advocacy for patients, communication and care coordination, decision-making, follow-through, knowledge of patients, and leadership), physician attitudes (doing more than the minimum required and responsibility), physician identity (main care provider), physician qualities (initiative), and quality of patient care (best and person-centered care) [19].

Resident duty hour restrictions have led to debate about the rise of a “shift-worker mentality” (i.e., the idea that the clock dictates a resident’s arrival and departure time, not the needs of the patient) and the loss of PCO [20]. As noted above, however, few studies have attempted to clarify the relationship between working hours and PCO among residents. A Japanese study explored the association of various personal or environmental factors with PCO [16]. Weekly working hours were included as an explanatory variable but no clear association between working hours and PCO was identified. Nevertheless, the study had three major limitations. First, the survey was conducted in 2021. As of early 2024, however, nearly all hospitals in Japan maintained strict management over physician working hours in preparation for implementation of the new regulations, due for that April. Second, the study was conducted under an exploratory study design; and third, the sample size was small. Accordingly, the association between working hours and PCO among residents needs to be re-examined in a hypothesis-testing study with a larger sample size in the context of the current era of physician work hour regulations.

Here, to better understand the association between working hours and PCO, we performed a nationwide cross-sectional study via a nationwide survey of medical residents in Japan.

## Methods

### Design, setting, and participants

This nationwide cross-sectional study formed a part of a research project regarding PCO [21]. It was conducted from January 17 to March 31, 2024 by distribution of an anonymous, online, self-administered questionnaire to early medical residents (i.e., postgraduate year 1 (PGY-1) and PGY-2 physicians, described in detail in the next paragraph) in Japan who had completed the General Medicine In-Training Examination (GM-ITE).

In the Japanese medical education system, medical trainees enter a two-year early residency program after graduating from their medical school and obtaining a medical license [22]. In this program, they must rotate through multiple clinical departments [22]. Upon completion of the early residency program, they progress to a three-to-five-year specialty training phase [22]. The GM-ITE was first conducted in 2011 by the Japan Institute for Advancement of Medical Education Program (JAMEP), a nonprofit organization, and over 50% of early medical residents throughout Japan now take it [21, 23, 24].

In this study, eligibility criteria included the GM-ITE examinees across Japan. Immediately following the GM-ITE, participants were asked to complete the questionnaire. Before participating, all participants were asked to read the research document which informed them of the anonymity and voluntary nature of the study. We included only participants who provided informed consent to participate.

### Measures

#### Outcome variable: PCO

We assessed the participants’ PCO using the Japanese version of the PCO Scale (J-PCOS) [25–27] (supplementary file). The J-PCOS has been published in 2021 in Japan [27]. Construct validity and internal consistency reliability of the scale were well examined in the validation study [27]. The scale has 13 items in 4 dimensions, namely assertiveness (6 items (e.g., “I was given the opportunity to make decisions independently about my patients’ care.”)), sense of ownership (2 items (e.g., “I felt a strong sense of ownership of my patients’ care.”)), diligence (2 items (e.g., “I personally made sure to go back and check that all orders were actually carried out.”)), and being the “go-to” person (i.e., being a reliable source of information and support for healthcare staff) (3 items (e.g., “I was the ‘go-to’ person for knowledge about my patients.”)) [27]. Each of the 13 items is rated on a 7-point Likert scale that ranges from 1 = strongly disagree to 7 = strongly agree.

In the present study, we employed the total score of the J-PCOS as primary outcome and the score for each domain of the J-PCOS as secondary outcome. Total score was calculated by averaging the responses to all 13 items. Domain scores were the mean of the respective domain. Both the J-PCOS total score and its domain scores ranged from 1 to 7, with greater scores indicating a higher PCO.

#### Explanatory variable: weekly working hours

The participants reported their weekly working hours, including weekday work duty, weekend work duty, and night emergency department duty, using a 10-category scale. In the analysis, we re-categorized the data of working hours into the following 7 groups: Category 1 (< 50 h), Category 2 (≥ 50 to < 55 h), Category 3 (≥ 55 to < 60 h), Category 4 (≥ 60 to < 70 h), Category 5 (≥ 70 to < 80 h), Category 6 (≥ 80 to < 90 h), and Category 7 (≥ 90 h). Because 60 h/week is generally the limit of working hours for physicians in Japan [10], we decided to set Category 4 as the reference group.

#### Covariates

We selected covariates for their possible relationship with working hours and PCO, with reference to previous studies [16, 25, 26, 28]. The covariates included sex (female or male), PGY (PGY-1 or -2), number of assigned inpatients (0–4, 5–9, 10–14, or ≥ 15), type of hospital (community hospital, university branch hospital, or university hospital), and size of hospital (< 400, 400–499, 500–699, or ≥ 700 beds).

### Statistical analysis

Data are reported using descriptive statistics, with categorical data reported as frequencies and percentages and continuous data as means and standard deviations.

To check whether multilevel analysis was needed, we assessed clustering effects by calculating the intraclass correlation coefficient (ICC) [29]. In our dataset, the ICC was less than 10% for the outcome variable. We therefore concluded that multilevel analysis was not required, and that we should use conventional analysis instead. We performed multivariable linear regression analysis with adjustment for possible confounders (sex, PGY, number of assigned inpatients, hospital type, and hospital size). We chose complete case analysis. A two-tailed *p* < 0.05 was considered statistically significant. According to our previous papers [30–32], the sample size was determined to be around 1500. For all statistical analyses, we used SPSS Statistics version 29.0 (IBM Corp).

### Ethical considerations

All participants in the study provided informed consent prior to participate. The study was approved by the Ethics Review Board of JAMEP (approval number: 23 − 21).

## Results

Of the 9106 participants who completed the GM-ITE, 2050 agreed to participate in the study. After excluding 214 with missing data, we analyzed the data of the remaining 1836 participants.

Table 1 demonstrates the characteristics of the participants. The most common option for average weekly working hours was “< 50 h” (Category 1, 395 (21.5%)), followed by “≥ 60 to < 70 h” (Category 4, 367 (20.0%)).

**Table 1:** Characteristics of the participants (*N* = 1836)

	Value
Sex, n (%)	
Female Male	576 (31.4) 1260 (68.6)
PGY, n (%)	
PGY-1 PGY-2	901 (49.1) 935 (50.9)
Number of assigned inpatients, n (%)	
0–4 5–9 10–14 ≥ 15	674 (36.7) 961 (52.3) 153 (8.3) 48 (2.6)
Type of hospital, n (%)	
Community hospital University branch hospital University hospital	1488 (81.0) 114 (6.2) 234 (12.7)
Size of hospital, n (%)	
< 400 beds 400–499 beds 500–699 beds ≥ 700 beds	511 (27.8) 389 (21.2) 561 (30.6) 375 (20.4)
Weekly working hours, n (%)	
< 50 h ≥ 50 to < 55 h ≥ 55 to < 60 h ≥ 60 to < 70 h ≥ 70 to < 80 h ≥ 80 to < 90 h ≥ 90 h	395 (21.5) 360 (19.6) 285 (15.5) 367 (20.0) 171 (9.3) 164 (8.9) 94 (5.1)
J-PCOS, mean (SD)	
Total score Assertiveness Sense of ownership Diligence Being the “go-to” person	4.84 (0.99) 4.85 (1.02) 5.06 (1.08) 4.89 (1.12) 4.62 (1.17)

Abbreviations: J-PCOS = Japanese version of the Patient Care Ownership Scale; PGY = postgraduate years; SD = standard deviation

Note: J-PCOS scores and dimension scores range from 1 to 7, with higher scores indicating higher PCO

Table 2 shows the results of the multivariable linear regression analysis that examined whether the number of working hours per week was associated with PCO. Medical residents who worked ≥ 70 to < 80 h/week (Category 5) or ≥ 80 to < 90 h/week (Category 6) had greater PCO than those working ≥ 60 to < 70 h/week (Category 4; reference group). Regarding individual PCO domains, assertiveness and being the “go-to” person showed a similar trend; working hours of ≥ 70 to < 80 h/week (Category 5) and ≥ 80 to < 90 h/week (Category 6) were associated with assertiveness and being the “go-to” person. Conversely, there was no clear trend in association between weekly working hours and sense of ownership, or between weekly working hours and diligence.

**Table 2:** Association of weekly working hours with PCO (*N* = 1836)

	Unadjusted mean difference (95% CI)	Adjusted^a^ mean difference (95% CI)
Total^b^		
Category 1 (< 50 h) Category 2 (≥ 50 to < 55 h) Category 3 (≥ 55 to < 60 h) Category 4 (≥ 60 to < 70 h) Category 5 (≥ 70 to < 80 h) Category 6 (≥ 80 to < 90 h) Category 7 (≥ 90 h)	-0.03 (-0.17 to 0.11) -0.05 (-0.19 to 0.10) 0.03 (-0.13 to 0.18) Ref. 0.21 (0.03 to 0.39)* 0.24 (0.06 to 0.42)** 0.25 (0.03 to 0.47)*	0.04 (-0.10 to 0.18) -0.04 (-0.18 to 0.10) 0.01 (-0.14 to 0.16) Ref. 0.20 (0.02 to 0.37)* 0.26 (0.08 to 0.44)** 0.17 (-0.05 to 0.40)
Assertiveness^b^		
Category 1 (< 50 h) Category 2 (≥ 50 to < 55 h) Category 3 (≥ 55 to < 60 h) Category 4 (≥ 60 to < 70 h) Category 5 (≥ 70 to < 80 h) Category 6 (≥ 80 to < 90 h) Category 7 (≥ 90 h)	-0.06 (-0.20 to 0.09) -0.06 (-0.20 to 0.09) 0.00 (-0.16 to 0.16) Ref. 0.20 (0.02 to 0.39)* 0.23 (0.04 to 0.42)* 0.26 (0.02 to 0.49)*	0.01 (-0.14 to 0.15) -0.06 (-0.20 to 0.09) -0.02 (-0.17 to 0.14) Ref. 0.19 (0.01 to 0.37)* 0.25 (0.07 to 0.44)** 0.18 (-0.05 to 0.42)
Sense of ownership^b^		
Category 1 (< 50 h) Category 2 (≥ 50 to < 55 h) Category 3 (≥ 55 to < 60 h) Category 4 (≥ 60 to < 70 h) Category 5 (≥ 70 to < 80 h) Category 6 (≥ 80 to < 90 h) Category 7 (≥ 90 h)	-0.13 (-0.28 to 0.03) -0.05 (-0.21 to 0.11) -0.00 (-0.17 to 0.16) Ref. 0.15 (-0.05 to 0.34) 0.18 (-0.02 to 0.38) 0.30 (0.06 to 0.55)*	-0.16 (-0.22 to 0.09) -0.03 (-0.19 to 0.12) -0.01 (-0.17 to 0.16) Ref. 0.14 (-0.06 to 0.33) 0.19 (-0.00 to 0.39) 0.20 (-0.05 to 0.45)
Diligence^b^		
Category 1 (< 50 h) Category 2 (≥ 50 to < 55 h) Category 3 (≥ 55 to < 60 h) Category 4 (≥ 60 to < 70 h) Category 5 (≥ 70 to < 80 h) Category 6 (≥ 80 to < 90 h) Category 7 (≥ 90 h)	0.12 (-0.14 to 0.18) -0.06 (-0.22 to 0.10) 0.07 (-0.10 to 0.24) Ref. 0.18 (-0.02 to 0.38) 0.19 (-0.02 to 0.40) 0.19 (-0.07 to 0.44)	0.09 (-0.07 to 0.25) -0.06 (-0.22 to 0.11) 0.06 (-0.12 to 0.23) Ref. 0.17 (-0.04 to 0.37) 0.20 (-0.01 to 0.40) 0.12 (-0.14 to 0.37)
Being the “go-to” person^b^		
Category 1 (< 50 h) Category 2 (≥ 50 to < 55 h) Category 3 (≥ 55 to < 60 h) Category 4 (≥ 60 to < 70 h) Category 5 (≥ 70 to < 80 h) Category 6 (≥ 80 to < 90 h) Category 7 (≥ 90 h)	0.06 (-0.11 to 0.23) -0.02 (-0.19 to 0.15) 0.07 (-0.12 to 0.25) Ref. 0.29 (0.08 to 0.50)** 0.35 (0.13 to 0.56)** 0.24 (-0.03 to 0.50)	0.13 (-0.04 to 0.29) -0.01 (-0.18 to 0.16) 0.06 (-0.12 to 0.24) Ref. 0.27 (0.07 to 0.48)* 0.37 (0.16 to 0.58)** 0.17 (-0.10 to 0.43)

Abbreviations: CI, confidence interval; J-PCOS, Japanese version of the Patient Care Ownership Scale; PCO, patient care ownership

^a^ Adjusted for sex, postgraduate years, the number of assigned patients, type of hospital, and size of hospital

^b^ All scores range from 1 to 7

* *p* < 0.05, ** *p* < 0.01

## Discussion

This nationwide cross-sectional study of medical residents in Japan elucidated the association between weekly working hours and PCO. Multivariable linear regression analysis showed that, compared with ≥ 60 to < 70 h/week, working hours of ≥ 70 to < 90 h/week were associated with greater PCO. The analysis also revealed that working hours of ≥ 70 to < 90 h/week were associated with assertiveness and being the “go-to” person, whereas there was no clear trend in associations between weekly working hours, sense of ownership, or diligence. In this era of working hour regulations, our findings will inform policymaking to determine appropriate resident work hours.

As noted in the Introduction, many researchers and clinical educators have expressed concerned that regulation of working hours may lead to the rise of a “shift-worker mentality” among medical residents [20]. Given our present findings, this concern appears both partly false but also partly true. Use of the term “shift-worker mentality” appears to emphasize the affective aspect of PCO [33]. This study showed that weekly working hours were not associated with sense of ownership, i.e., affective aspect of PCO. Conversely, while the concept of PCO is used to emphasize the affective aspect, it has been expanded in recent years to a multifaceted concept which includes both cognitive and behavioral aspects [28, 34]. Our present findings revealed that weekly working hours of ≥ 70 to < 90 h were associated with assertiveness and being the “go-to” person, which means that medical residents working longer than the general limit for physicians in Japan (i.e. 60 h/week) had greater experience of these dimensions of PCO. Therefore, our findings suggest the need to take a comprehensive view of PCO, which includes not only its emotional-affective aspect but also a cognitive-behavioral aspect. In other words, it is time to reconceive PCO beyond the bounds of “shift-worker mentality.”

Regulations limiting physician working hours were implemented in April 2024, with the initial expectation that weekly working hours would be capped at 80 h. From the perspective of PCO, however, our present findings suggest that an 80-hour work week will not necessarily yield the best results. This finding is significant as it suggests the potential for future interventions in the training environment, including consideration of working hours. Our findings suggest that a certain amount of time may be required to nurture PCO, and that, in terms of PCO training, working hours of 80–90 h/week may be an option. Nevertheless, concerns remain that excessively long working hours can lead to poor mental health. Numerous studies have indicated that an 80-h-per-week limit is likely to have a desirable effect on mental health [29]. In their systematic review, Fletcher et al. showed that five of eight studies examining the association between the 2003 duty hour rules (i.e., 80-h/week limit) and resident wellness in the U.S. reported decreased levels of burnout [35]. Another U.S. study reported that working > 80 h/week corresponded to burnout rates of approximately 70% versus around 40% for < 80 h/week [36]. In addition, many other factors need to be considered in determining appropriate working hours, including quality of patient care and resident education. Considering the above, there is room for further discussion on whether it is better to limit working hours uniformly as a group or to set them based more on individualized characteristics, such as individual clinical performance, mental health, PCO, etc.

To our knowledge, this is the first study to investigate the association between weekly working hours and PCO under a hypothesis-testing study design in the context of the present era of physician work hour regulation in Japan. Our data were based on data from the nationwide JAMEP network, which covered a wide range of medical residency training programs. Furthermore, PCOS is an established tool for assessing PCO [25–27]. Accordingly, the results of this study have relatively high external validity. Nevertheless, our study was conducted in a single country, whereas working hours and PCO appear to be influenced by culture and system, which vary among countries. Accordingly, comparison of our study with similar studies outside Japan would likely prove insightful in better understanding the relationship between working hours and PCO.

We note some potential limitations of this study. First, since it was conducted under a cross-sectional design, it did not allow us to determine causality or the direction of relationships between working hours and PCO. Additional longitudinal research is needed to confirm these. Second, our data for weekly working hours were based on self-reported information. However, a previous study indicated the relative accuracy of working hours self-reported by resident physicians [37], which ameliorates our concern. Third, the response rate to our questionnaire was relatively low. It is possible that medical residents with shorter working hours per week or poorer PCO were less likely to complete the questionnaire. If so, this could result in underestimation of the association between weekly working hours and PCO. Fourth, the proportion of residents who worked in a university hospital was less than 20%. Given that approximately half of resident physicians in Japan are affiliated with a university hospital, this may have impacted the overall results. This point should be considered when interpreting the findings. Fifth, although the study results were statistically significant, the educational relevance of the score difference remains unknown. Further research to interpret J-PCOS scoring may clarify the practical significance of the results of this study. Sixth, although we identified covariates based on the previous literature, it is possible that there are unknown confounding factors. Individual patient and physician characteristics may influence the results.

## Conclusions

In terms of PCO training, the working hours of 80–90 h/week may be an option, though a thorough evaluation of various factors is necessary to determine the optimal working hours of resident physicians. The divergence in the results of the PCO subscale analysis indicates the need to capture PCO from a comprehensive perspective. A comparison of our study with similar studies conducted outside of Japan would provide valuable insights into the association between working hours and PCO.

## Electronic supplementary material

Below is the link to the electronic supplementary material.

Supplementary Material 1

## Data Availability

Only upon reasonable request, the corresponding author can provide the data sets generated and analyzed in this study.

## Abbreviations

GM-ITE: General Medicine In-Training Examination
ICC: Intraclass correlation coefficient
J-PCOS: Japanese version of the Patient Care Ownership Scale
JAMEP: Japan Institute for Advancement of Medical Education Program
PCO: Patient care ownership
PGY: Postgraduate year
