# Supplementary material for: Association between residents’ work hours and patient care ownership: a nationwide cross-sectional study in Japan
Source: BMC Med Educ. 2025 Mar 15;25:385. doi: 10.1186/s12909-025-06941-1 (PMC11909906; doi:10.1186/s12909-025-06941-1)
Supplement: Supplementary file 1 — Supplementary Material 1 [file 12909_2025_6941_MOESM1_ESM.docx]

**Supplementary file: the Japanese version of the Patient Care Ownership Scale**

| **Item^a^** | **Factor^b^** |
| --- | --- |
| Q1. I was vocal and assertive about my patients’ best treatment/care. | Factor 1 |
| Q2. I was the “go-to” person for knowledge about my patients. | Factor 4 |
| Q3. I was proactive in checking up on my patients, rather than being called with questions or concerns. | Factor 4 |
| Q4. I ensured good continuity of care even when I was absent from the service. | Factor 1 |
| Q5. I felt comfortable telling the attending what I felt was the right thing to do for my patients, rather than just letting them decide. | Factor 1 |
| Q6. I made sure that the nursing staff was updated with the day’s plan. | Factor 4 |
| Q7. I was given the opportunity to make decisions independently about my patients’ care. | Factor 1 |
| Q8. I personally made sure to go back and check that all orders were actually carried out. | Factor 3 |
| Q9. When carrying out my patient’s management plan, I took extra care to make sure that things did not fall through the cracks. | Factor 3 |
| Q10. I felt comfortable making decisions independently about my patients’ care. | Factor 1 |
| Q11. I challenged the team as needed if I felt it was in my patients’ best interest, no matter how much push back I got. | Factor 1 |
| Q12. I felt responsible for my patients’ care, even after my shift ended. | Factor 2 |
| Q13. I felt a strong sense of ownership of my patients’ care. | Factor 2 |

^a^ Rated on a seven-point Likert scale: 1 = Strongly disagree; 2 = Disagree; 3 = Somewhat disagree; 4 = Neither agree nor disagree; 5 = Somewhat agree; 6 = Agree; 7 = Strongly Agree

^b^ Factor 1 = Assertiveness; Factor 2 = Sense of ownership; Factor 3 = Diligence; Factor 4 = Being the “go-to” person
